# Supplementary material for: Combination of Irinotecan and Melatonin with the Natural Compounds Wogonin and Celastrol for Colon Cancer Treatment
Source: Int J Mol Sci. 2023 May 31;24(11):9544. doi: 10.3390/ijms24119544 (PMC10253823; doi:10.3390/ijms24119544)
Supplement: Supplementary file 1 [file ijms-24-09544-s001.zip › ijms-2391395-supplementary.pdf]

Supplement 1 - Flow cytometry results.

LOVODX

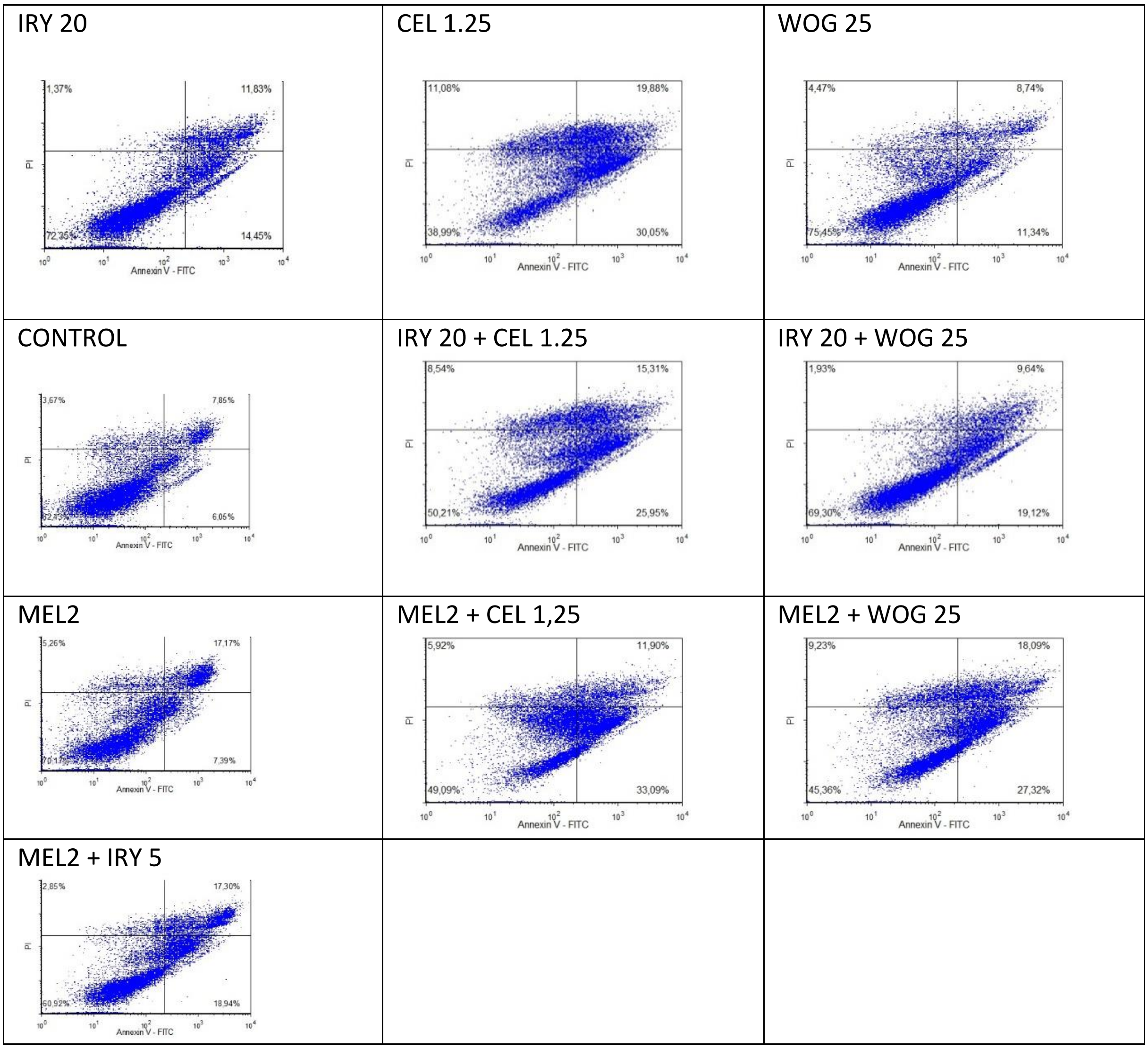

Figure 1 The representative examples of flow cytometry results for effects of 72 hours of incubation of irinotecan (5 µM and 20 µM), melatonin (2 mM), wogonin (25 µM) and celastrol (1.25 µM) with LOVO/DX cells on the frequency of apoptosis and necrosis. The cells were double stained with Annexin V- Alexa Fluor®488 and PI fluorescent dyes (Alexa Fluor® 488 Annexin V/ Dead Cell Apoptosis Kit) and analyzed by flow cytometry. The results are presented as a percentage of apoptotic cells (Annexin V- Alexa Fluor®488+ and PI- or Annexin V- Alexa Fluor®488+ and PI+) and necrotic cells (Annexin V- Alexa Fluor®488- and PI+). Abbreviations: Iry (irinotecan), Mel (melatonin), Cel (celastrol), Wog (wogonin).

LOVO

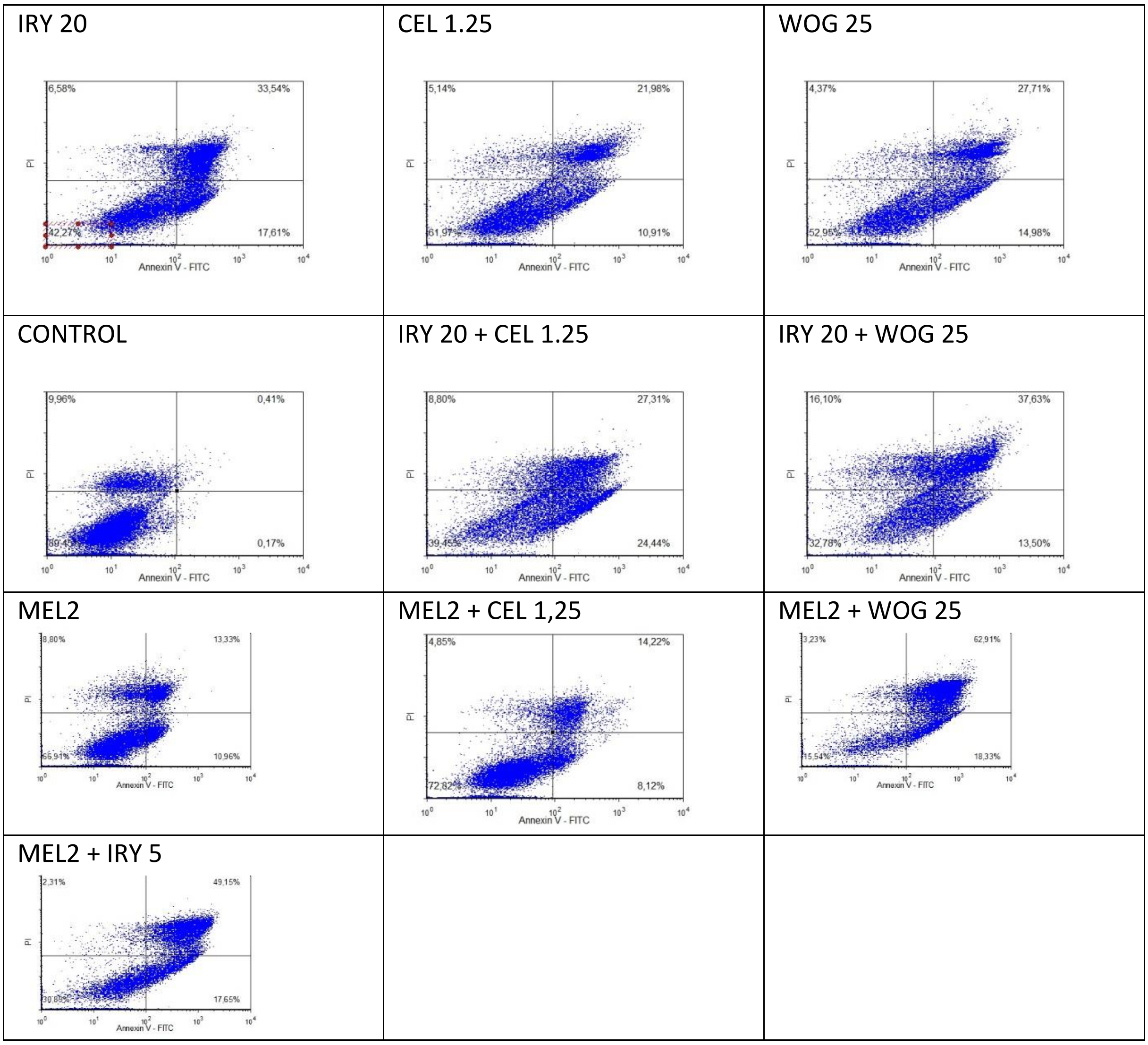

Figure 2 The representative examples of flow cytometry results for effects of 72 hours of incubation of irinotecan (5 µM and 20 µM), melatonin (2 mM), wogonin (25 µM) and celastrol (1.25 µM) with LOVO cells on the frequency of apoptosis and necrosis. The cells were double stained with Annexin V- Alexa Fluor®488 and PI fluorescent dyes (Alexa Fluor® 488 Annexin V/ Dead Cell Apoptosis Kit) and analyzed by flow cytometry. The results are presented as a percentage of apoptotic cells (Annexin V- Alexa Fluor®488+ and PI- or Annexin V- Alexa Fluor®488+ and PI+) and necrotic cells (Annexin V- Alexa Fluor®488- and PI+). Abbreviations: Iry (irinotecan), Mel (melatonin), Cel (celastrol), Wog (wogonin).
